# Strength Training and Nutrition Help Prevent Sarcopenia in Older Adults

**DOI:** 10.3390/ijerph22071118

**Published:** 2025-07-16

**Authors:** Milton Pereira, Ana Carolina Silva, Vinícius Mapa, Lilian Peixoto, Ingrid Lacerda, João Batista Ferreira-Júnior, Izinara Rosse, Emerson Cruz de Oliveira, Lenice Kappes Becker, Gabriela Venturini, Daniel Barbosa Coelho

**Affiliations:** 1 School of Physical Education, Physiotherapy and Occupational Therapy, Federal University of Minas Gerais, Belo Horizonte 31270-901, MG, Brazil; milton.efi2020@gmail.com; 2 Postgraduate Program in Health and Nutrition, Federal University of Ouro Preto, Ouro Preto 35400-000, MG, Brazil; anacs.nutricao@gmail.com (A.C.S.); lilian.lopes@aluno.ufop.edu.br (L.P.); ingridfisionobre@gmail.com (I.L.); 3 School of Physical Education and Postgraduate Program in Health and Nutrition, Federal University of Ouro Preto, Ouro Preto 35400-000, MG, Brazil; viniciuscm6@gmail.com (V.M.); emerson@ufop.edu.br (E.C.d.O.); lenice@ufop.edu.br (L.K.B.); 4 Department of Physical Education, Federal Institute of Southeast MG—Campus Rio Pomba, Rio Pomba 36180-000, MG, Brazil; jbfjunior@gmail.com; 5 School of Pharmacy, Federal University of Ouro Preto, Ouro Preto 35400-000, MG, Brazil; izinara.cruz@ufop.edu.br; 6 Federal Center for Technological Education (CEFET MG), Leopoldina Campus, Leopoldina 36700-000, MG, Brazil; doutoragabrielaventurini@gmail.com

**Keywords:** sarcopenia, resistance training, nutrition

## Abstract

Sarcopenia is a musculoskeletal, progressive, and generalized disease characterized by decreased muscle strength and mass, leading to reduced quality of life. Sarcopenia is directly related to age, a sedentary lifestyle, and poor nutrition. The objective of this study was to evaluate the effect of 12-week progressive intensity Resistance Training (RT) associated with nutritional advice on the frequency of sarcopenia in older adults. A total of 74 older adults (37 in the intervention group and 37 in the control group), with a mean age of 69.1 ± 6.85 years, were included in the study. The sarcopenia status of the participants was assessed at baseline and after a 12-week intervention. In the intervention group, resistance training combined with nutritional counseling reduced the prevalence of sarcopenia from 35.14% to 0% (*p* < 0.001). Additionally, participants in the intervention group showed significant improvements in handgrip strength (from 27.70 ± 10.71 to 30.24 ± 10.38 kg), chair stand test performance (from 14.04 ± 3.46 to 11.67 ± 1.80 s), and time up and go test (from 7.49 ± 1.20 to 6.74 ± 0.95 s) (*p* < 0.05). On the other hand, the control group increased the incidence of sarcopenia (*p* < 0.001). After 12 weeks, progressive intensity RT associated with nutritional advice proved to be an effective treatment to reverse sarcopenia and help participants remain non-sarcopenic. In addition, the results of this study provide information about efficient and non-pharmacological sarcopenia treatment.

## 1. Introduction

The life expectancy of the population has increased considerably in recent decades [1]. The exponential population increase requires active and healthy aging, mainly maintaining the autonomy and participation of the older population in society [1]. The aging process leads to natural and progressive muscle mass reduction, central nervous system damage, decreased anabolic pathways, and increased low-grade chronic inflammation [2].

Associated with aging, other factors such as sedentary lifestyle, poor nutrition, bad lifestyle habits, and the involvement of other non-communicable chronic diseases, such as sarcopenia, can affect the autonomy and independence of older people [3,4]. Sarcopenia is a progressive and generalized musculoskeletal disorder characterized by decreased strength and muscle mass, with decreased physical performance in the most severe stages of the disease [3].

Sarcopenia decreases the quality of life of older people, increasing the risk of falls and fractures, morbidity and mortality, and causing several physical, social, and economic consequences [5,6]. Ethgen [7] estimated an increased prevalence of sarcopenia between 60 and 70% in 28 European countries by the year 2045 and that 22.3% of the population over 65 years will be sarcopenic. In addition to other associated diseases, physical inactivity and nutritional status are directly related to the prevalence of sarcopenia [3,8].

Resistance training (RT) has been an effective, applicable, and practical non-pharmacological treatment to prevent sarcopenia [4,9,10]. The combination of RT and nutritional advice, especially adequate protein and essential amino acid intake, has promoted positive protein balance [5].

According to the most recent ESPEN guidelines, older adults should consume between 1.0 and 1.5 g/kg/day of protein, depending on their nutritional status, health condition, and physical activity level [11]. In cases of acute or chronic illness, or severe malnutrition, intake may be increased up to 2.0 g/kg/day, under clinical supervision [11].

Despite growing recognition of the role of nutrition and exercise, there is still limited evidence on the combined effect of individualized nutritional counseling and progressive resistance training on sarcopenia outcomes in community-dwelling older adults. Most previous studies have tended to focus on either exercise or dietary intervention alone, and few have assessed their synergistic impact in real-world, non-institutionalized populations.

Therefore, considering the several physical, social, and economic consequences of sarcopenia, the objective of this study was to evaluate the effect of 12-week progressive intensity RT associated with nutritional advice on the frequency of sarcopenia in older people.

## 2. Materials and Methods

The project was approved by the Human Research Ethics Committee of the Federal University of Ouro Preto (Resolution 466/2012) under CAAE 28686920.8.0000.5150. The volunteers signed the Free and Informed Consent Form informing about risks and benefits, privacy and access to information obtained during the research for the participant’s knowledge.

### 2.1. Experimental Design

This was a prospective study in which anthropometric measurements and sarcopenia status were assessed at baseline and after 12 weeks of intervention. Volunteers for the intervention group were recruited through posters placed at the Federal University of Ouro Preto and nearby community facilities. The control group consisted of 37 older adults participating in a local project aimed at promoting quality of life and recreational activities. The control group was advised to maintain their usual routines throughout the 12-week period, without engaging in any structured exercise or receiving nutritional guidance. Sarcopenia diagnostic criteria, including assessments of muscle strength, muscle mass, and physical performance, were applied to both groups at baseline and after the 12-week period.

### 2.2. Sample

Participants were recruited through public advertisements, including posters, flyers, and informational talks, placed in local health centers, community centers, university facilities, and social clubs aimed at older adults in Ouro Preto, Brazil. The recruitment specifically invited individuals aged 50 years and older to participate in a study on muscle health and aging. All interested volunteers underwent initial screening, including anthropometric measurements and functional assessments, to determine sarcopenia status based on EWGSOP2 criteria [3].

Sample size calculation was performed using the formula for paired group comparisons (Student’s *t*-test for dependent samples), based on a significance level of 5% (α = 0.05), statistical power of 80% (β = 0.20), a mean difference of 1.64 s, and a standard deviation of 2.04 s for the Chair Stand Test, according to data from Fachineto et al. [12]. The calculation indicated that a minimum of 12 participants would be required per group. However, considering possible dropouts and to strengthen statistical validity, we included 37 participants in the intervention group and 37 in the control group.

Inclusion criteria were: (1) age ≥ 50 years; (2) ability to perform the functional and diagnostic tests for sarcopenia; (3) no contraindications for resistance training or dietary modifications; and (4) agreement to participate in the 12-week intervention protocol.

Exclusion criteria included: (1) major surgery in the last 12 months; (2) cardiovascular, neurological, orthopedic or metabolic diseases, including type 2 diabetes, metabolic syndrome, or severe dyslipidemia; (3) current use of anabolic steroids or protein supplements; (4) recent participation in structured physical training programs; and (5) significant cognitive impairment as judged by clinical screening.

For the final analysis, only participants in the intervention group who attended at least 70% of the training sessions (i.e., ≥25 of 36 sessions) were included.

### 2.3. Anthropometric Assessment

The anthropometric assessment consisted of body mass (BM) and height measurements, for subsequent calculation of the body mass index (BMI). Measurements of triceps, biceps, subscapular, and suprailiac skinfolds were taken for later calculation of the total body fat percentage (TBF).

For the determination of BM (kg), a digital scale (Filizola^®^, São Paulo, SP, Brazil) with a precision of 0.02 kg, previously calibrated, was used. Height (meters) was measured using a stadiometer with a precision of 0.5 cm (SUNNY^®^, São Paulo, SP, Brazil). To determine the BMI, the formula was used: BMI = MC/EST^2^.

Skinfolds were measured using a picometer with a reading range of 85 mm, sensitivity equal to 0.1 mm, and pressure of 10 g/mm^2^ (Cescorf^®^, Porto Alegre, RS, Brazil) [13]. To estimate the TBF, the equation of the sum of the four skinfolds was used, according to Durnin & Womersley [14].

### 2.4. Assessment of Sarcopenia Status

To assess muscle strength, the following tests were used: Hand Grip Strength (HGS) using a hydraulic hand dynamometer (Jamar^®^, Tupã, SP, Brazil), and the 5-times CST, which consists of standing up and sitting on a chair. The adopted cut-off points were <27 kg for men and <16 kg for women for HGS, and for CST, it was >15 s [3]. Subjects with a low muscle strength were classified as probably sarcopenic.

Muscle mass was determined by the bioimpedance method (Biodynamics TBW 310e^®^, Macon, GA, United States of America). The absolute muscle mass (Kg) was normalized for height (muscle mass (Kg)/height (m^2^) and named Muscle Mass Index (MMI). The cut-off point adopted to classify sarcopenia was muscle mass < 20 kg or MMI < 7.0 kg/m^2^ for men and muscle mass < 15 kg or <5.5 kg/m^2^ for women [3]. Subjects with a low muscle mass were classified as sarcopenic.

To evaluate the physical performance, the Gait Speed (GS) and Time Up and Go Test (TUGT) tests were used. The cut-off point adopted in the GS test was a speed ≤ 0.8 m/s [3]. The cut-off point adopted for the TUGT test was ≥20 s [3]. The volunteers performed three attempts before the start of each test to familiarize themselves with the tests. Subjects with a low physical performance were classified as sarcopenic.

Based on the results of the diagnostic tests, the study participants were classified as non-sarcopenic (with normal upper and lower limb muscle strength), probably sarcopenic (low upper and/or lower limb muscle strength), sarcopenic (low muscle strength and low muscle mass), or severely sarcopenic (low muscle strength, low muscle mass and low physical performance and/or functional capacity) [3]. All study subjects who were classified as probably sarcopenic, sarcopenic, and severely sarcopenic were classified as sarcopenic to form two groups for comparison, as recommended by Goltz [15].

### 2.5. Resistance Training

The RT was performed three times a week, on non-consecutive days, at the Bodybuilding Laboratory of the Physical Education School of the Federal University of Ouro Preto (EEF—UFOP). The volunteers trained until they completed 36 workouts (12 weeks). Before the beginning of the intervention, two weeks of familiarization with the exercises and devices were performed, with low intensity.

After the familiarization with exercises, the application of the prediction test of a maximum repetition (1-RM) [16] was performed, which consisted of each volunteer performing 10 maximum repetitions to estimate 1-RM. After the tests, a period of 12 weeks of RT with progressive intensity (60% to 85% 1-RM) was performed.

In the 1st and 2nd weeks, the volunteers trained with a load of 60% of 1-RM, and a volume of 3 sets of 12 to 15 repetitions; in the 3rd and 4th weeks, with 70% of 1-RM, 3 sets of 10 to 12 repetitions; on the 5th and 6th week, with 80% of 1-RM, 3 sets of 8 to 10 repetitions, and from the 7th week, the load was 85% of 1-RM and the volume, 3 sets of 6 to 8 repetitions, until the 36 training sessions of the intervention were completed.

The interval between series was 60 s and the repetition duration in the eccentric and concentric phases was 2 s each. A supervisory ratio of 4:1 (1 experienced monitor for every 4 volunteers) was established. The exercises performed were: anterior pulldown (supinated), alternating curls, seated rowing, high pulley triceps, bench press (barbell or apparatus), leg curls, leg extensions, oblique abdominals, infra-abdominals (hands-on chest), soleus bench, and free squat with a dumbbell [17,18].

Each resistance training session lasted approximately 60 min. Adherence was monitored throughout the intervention, and only participants who attended at least 70% of the sessions (i.e., a minimum of 25 out of 36 sessions) were included in the analysis. All included participants completed the protocol without adverse events.

### 2.6. Nutritional Advice

At the beginning of the intervention, after the initial dietary assessment, participants received individualized nutritional counseling alongside the resistance training protocol. The counseling focused primarily on promoting healthy eating habits and encouraging adequate protein intake. Emphasis was placed on the consumption of high-biological-value protein sources (e.g., dairy products, eggs, fish, lean meats, and legumes) and the proper distribution of protein throughout the day [11,19].

Nutritional guidance was delivered verbally and in printed form by a dietitian at the start of the training program and was reinforced verbally on a weekly basis throughout the 12 weeks of intervention. The effectiveness of the nutritional counseling was evaluated by assessing dietary protein intake using 24 h dietary recalls (24HR), applied before and after the intervention [11,19]. Recalls were conducted individually by trained professionals using standardized portion estimation tools and the national food composition table. The intake data were analyzed and expressed in grams of protein per kilogram of body weight per day (g/kg/day) [11,19].

In addition to general nutritional recommendations, the counseling was strategically tailored to the needs of sarcopenic individuals. The food pyramid and NOVA classification were used as starting points to explain food quality and prioritize minimally processed protein-rich foods. Participants were guided to incorporate protein sources into each main meal, including practical examples based on their typical dietary patterns and socioeconomic context. Recipes and suggestions for combining plant and animal proteins (e.g., rice and beans, eggs with vegetables, milk with oats) were provided, emphasizing affordable and accessible options. The counseling also addressed meal timing, encouraging protein intake shortly after resistance training sessions to support muscle protein synthesis [11,19].

### 2.7. Statistical Analysis

The categorical variables are presented as absolute and relative frequencies. Descriptive data are expressed as mean ± standard deviation for normally distributed variables and as median (minimum–maximum) for non-normal distributions. Levene’s test was used to assess homogeneity of variance for age between groups. The Pearson’s chi-square test was applied to compare sarcopenia status changes between the control and intervention groups after the 12-week intervention, with Cramer’s V used to assess effect size.

To evaluate changes in functional performance (handgrip strength, chair stand test, gait speed, and Timed Up and Go), skeletal muscle mass, muscle mass index, and protein intake within groups, paired-sample *t*-tests were used for normally distributed variables, while the Wilcoxon signed-rank test was applied to non-parametric data. Between-group comparisons of gender distribution were also performed using Pearson’s chi-square test. All statistical tests were two-tailed and conducted with a significance level of 5% (*p* < 0.05).

Statistical analyses were performed using IBM SPSS Statistics software version 20 (IBM Corp., Armonk, NY, USA) and the JAMOVI statistical software (version 2.6.26) for specific paired comparisons and effect size calculations.

## 3. Results

### 3.1. Characteristics of the Sample

Table 1 presents the anthropometric characteristics of participants in the intervention and control groups over the 12-week period.

The distribution of gender did not differ significantly between the intervention and control groups (Table 2). In the intervention group, females represented 56.8% and males 43.2%; in the control group, females accounted for 62.2% and males 37.8%. No statistically significant difference was found (χ^2^(1) = 0.282, *p* = 0.595; Fisher’s exact test = 0.625), indicating a balanced gender distribution at baseline.

Table 3 presents the sarcopenia status between control and intervention groups before intervention.

### 3.2. Diagnostic Parameters of Sarcopenia

After 12 weeks of intervention (Table 4), participants in the intervention group demonstrated improvements in handgrip strength (HGS), chair stand test (CST), and Timed Up and Go (TUGT) performance after the intervention. Specifically, HGS increased significantly (*p* = 0.002), while CST and TUGT times decreased (*p* < 0.001), indicating improved functional performance. No statistically significant changes were found in skeletal muscle mass (MME), muscle mass index (IMM) and Gait Speed Test (GS).

In the control group (Table 5), handgrip strength (HGS), chair stand test (CST), and Timed Up and Go (TUGT) performance showed statistically significant changes over the 12-week period (*p* < 0.01). HGS values decreased, indicating a decline in muscle strength, while CST and TUG times increased, suggesting impaired lower-limb function and mobility. No significant changes were observed in skeletal muscle mass (MME), muscle mass index (IMM), or gait speed (VM) (*p* > 0.05).

### 3.3. Nutritional Outcomes: Protein Intake

In the intervention group, protein intake increased significantly after the 12-week intervention (Table 6). Mean intake rose from 0.99 ± 0.24 g/kg/day at baseline to 1.22 ± 0.30 g/kg/day post-intervention (*p* < 0.001), with a large effect size (Cohen’s d = −0.916).

### 3.4. Changes in Sarcopenia Status

Table 7 presents the sarcopenia status changes between control and intervention groups after intervention. There was a significant difference regarding sarcopenia status changes after 12 weeks between the intervention and control groups (X^2^ (3) = 47.867; *p* < 0.001). The Cramer’s V post-test (Crammer’s V = 0.804; *p* < 0.001) indicates the effect size of the volunteers in the intervention group. There was an increased prevalence of sarcopenia in the control group.

## 4. Discussion

In the present study, the progressive intensity 12-week RT associated with nutritional advice decreased the frequency of sarcopenia in older people. The intervention showed a positive effect, with 64.86% of participants remaining non-sarcopenic and 35.13% becoming non-sarcopenic. In the control group, 45.95% of participants maintained the status of sarcopenia, with 32.43% of participants transitioning to sarcopenia.

These results corroborate del Campo Cervantes [20], who reported a reduction from 47.4% to 33.3% in the status of severe sarcopenia in older participants after a 12-week RT program. In addition to RT, those participants ingested 0.9 g of protein/kg/day and followed a diet of 1557 kcal/day, which may partly explain the impact of the intervention, since the proteins and amino acids provided in the diet are necessary for the synthesis of muscle proteins and to maintain a positive nitrogen balance [21,22]. On the other hand, another study reported that 12-week RT combined with supplementation of 30 g protein/day (1.2 g/kg/day) showed no significant improvement in sarcopenia diagnostic parameters (grip strength, muscle mass, and physical performance) [23]. This highlights that not only protein quantity, but also nutrient timing, food quality, and dietary patterns influence muscle adaptation [21,23,24].

In the present study, although the volunteers received no supplementation, they were instructed about the food groups according to the NOVA food processing classification [19], food pyramid, classification and principles of healthy eating. The counseling emphasized adequate protein intake and distribution throughout the day [5,19]. As a result, mean protein intake in the intervention group increased significantly from 0.99 ± 0.24 to 1.22 ± 0.30 g/kg/day (*p* < 0.001), aligning with current ESPEN recommendations for older adults [19]. This demonstrates that individualized food-based interventions can be effective and feasible in promoting meaningful dietary improvements [25]. This is noteworthy in real-world settings where supplement use may not be feasible and highlights the impact of targeted behavioral interventions on improving protein intake adequacy in older adults [25].

Beyond sarcopenia prevalence, participants in the intervention group showed significant improvements in key physical performance outcomes. Increases in handgrip strength and reductions in chair stand and TUG test times indicate gains in strength and functional mobility. However, no significant changes were observed in muscle mass (MME), muscle mass index (IMM), or gait speed. These findings suggest that the intervention led to neuromuscular adaptations, such as improved motor unit recruitment and efficiency, prior to measurable hypertrophy, a response commonly seen in early phases of resistance training among older adults [26]. Such adaptations are particularly relevant in older adults, where early improvements from resistance training tend to reflect efficiency rather than hypertrophy.

The main limitation of this study is the absence of dietary intake data from the control group, which limited comparisons of dietary behavior between groups. Although the intervention group demonstrated significant improvements in protein intake, it was not possible to evaluate whether diet contributed to the sarcopenia progression in the control group. Despite this, the results reinforce the utility of non-pharmacological strategies for sarcopenia treatment based on resistance training and nutritional guidance.

Although the diagnostic cut-off points for sarcopenia were applied according to gender-specific EWGSOP2 criteria, statistical analyses were not stratified by gender due to the limited sample size, which reduced statistical power. However, the groups were balanced at baseline, minimizing the risk of confounding related to gender distribution.

However, RT associated with nutritional advice, mainly daily intake and adequate distribution of proteins, reduced the frequency of sarcopenia by 35.13% and maintained the non-sarcopenic status in 64.86% of the participants in the intervention group. According to the results of the present study, RT associated with nutritional advice is an effective, applicable, and practical treatment to reduce sarcopenia and promote functional improvements.

## 5. Conclusions

The results of this study provide information about efficient and non-pharmacological sarcopenia treatment. In conclusion, a 12-week progressive resistance training program combined with nutritional advice completely reduced sarcopenia prevalence in older adults.

## Figures and Tables

**Table 1:** Characterization of the participants in the intervention and control groups.

	Age (Years)	BMI (kg/m^2^)	MMI (kg/m^2^)	TBF (%)
Control (37)	69.81 ± 6.65	28.48 ± 5.36	7.99 ± 1.61	38.30 ± 5.66
Intervention (37)	64.27 ± 7.06	28.14 ± 4.56	8.92 ± 1.61	38.09 ± 7.42

Values expressed as mean and standard deviation; BM = body mass; BMI = body mass index; MMI = muscle mass index; TBF = total body fat.

**Table 2:** Gender distribution according to sarcopenia status between control and intervention groups at baseline.

Gender	Intervention	Sarcopenic	Total
Female	21 (56.8%)	23 (62.2%)	44 (59.5%)
Male	16 (43.2%)	14 (37.8%)	30 (40.5%)

Values are presented as absolute frequency and percentage. Statistical comparison was performed using Pearson’s chi-square test. No significant difference was found in gender distribution between groups (χ^2^(1) = 0.282, *p* = 0.595; Fisher’s exact test = 0.625).

**Table 3:** Sarcopenia status between the control and intervention groups at baseline.

	Non-Sarcopenic	Sarcopenic	Total
Control	18	19	37
(%)	48.65	51.35	100
Intervention	24	13	37
(%)	64.86	35.14	100

Values expressed as absolute and relative frequency.

**Table 4:** Functional and muscle parameters before and after 12 weeks in the intervention group.

	Pre-Intervention (37)	Post-Intervention (37)	*p*-Value	Cohen’s D
HGS (kg)	26 (9–54)	28 (14–55) *	0.002	−0.6437
CST (s)	13.39 (9.07–21.18)	11.50 (8.04–14.95) *	<0.001	0.8094
Muscle Mass (kg)	22.50 (13.28–35.30)	22.21 (13.70–34.47)	0.781	0.0556
Muscle Mass Index (kg/m^2^)	9.07 (6.02–11.77)	8.97 (6.01–12.07)	0.762	0.0603
GS (m/s)	1.43 (0.82–2.05)	1.39 (0.82–2.42)	0.720	0.0697
TUGT (s)	7.25 (5.37–11.25)	6.47 (5.33–8.83) *	<0.001	0.7710

Values are expressed as median (minimum–maximum). *p*-values refer to Wilcoxon signed-rank tests comparing pre- and post-intervention scores within the intervention group. Interpretation of the direction of change depends on the clinical meaning of the variable. *: Statistically significant results (*p* < 0.05).

**Table 5:** Functional and muscle parameters before and after 12 weeks in the control group.

	Pre-Intervention (37)	Post-Intervention (37)	*p*-Value	Cohen’s D
HGS (kg)	24.19 ± 6.06	16.92 ± 3.91 *	<0.001	1.739
CST (s)	15.60 ± 2.57	17.78 ± 3.72 *	<0.001	−0.668
Muscle Mass (kg)	19.22 ± 4.69	19.10 ± 4.69	0.157	0.270
Muscle Mass Index (kg/m^2^)	7.42 ± 2.07	7.37 ± 2.02	0.160	0.266
GS (m/s)	1.40 ± 0.29	1.45 ± 0.26	0.232	–0.200
TUGT (s)	9.40 ± 2.34	10.77 ± 2.26 *	<0.001	–0.676

Values are expressed as mean ± standard deviation. *p*-values refer to Student’s *t*-tests comparing pre- and post-intervention scores within the control group. Interpretation of the direction of change depends on the clinical meaning of the variable. *: Statistically significant results (*p* < 0.05).

**Table 6:** Daily protein intake before and after 12 weeks in the intervention group.

	Pre-Intervention (37)	Post-Intervention (37)	*p*-Value	Cohen’s D
Protein intake (g/kg/day)	0.99 ± 0.24	1.22 ± 0.30 *	<0.001	−0.916
% of participants with intake ≥ 1.0 g/kg/day	40.5%	78.4%	-	-
% of participants with intake between 1.2 and 2.0 g/kg/day	18.9%	40.5%	-	-
Intake range (min–max) (g/kg/day)	0.51–1.55	0.81–1.91	-	-

Values are expressed as mean ± standard deviation. A paired Student’s *t*-test was used to compare protein intake before and after the intervention in the intervention group. Negative Cohen’s d values indicate improvement when higher scores represent better outcomes. *: Statistically significant (*p* < 0.001).

**Table 7:** Sarcopenia status between the control and intervention groups after 12 weeks.

	Remained Non-Sarcopenic	Non-Sarcopenic to Sarcopenic	Sarcopenic to Non-Sarcopenic	Remained Sarcopenic	Total
Control	6	12	2	17	37
(%)	16.21	32.43	5.41	45.95	100
Intervention	24 *	0 *	13 *	0 *	37
(%)	64.86 *	0 *	35.13 *	0 *	100

Values expressed as absolute and relative frequency. *: Significant difference between intervention and control groups after 12 weeks (*p* < 0.001).

## Data Availability

Data is unavailable due to privacy or ethical restrictions.

## Abbreviations

The following abbreviations are used in this manuscript:

RT	Resistance Training
TBF	Total Body Fat
BM	Body Mass
BMI	Body Mass Index
MMI	Muscle Mass Index
TUGT	Time Up and Go Test
HGS	Hand Grip Strength
CST	Chair Stand Test
GS	Gait Speed
